# Extraction of polyphenols and synthesis of new activated carbon from spent coffee grounds

**DOI:** 10.1038/s41598-019-54205-y

**Published:** 2019-11-27

**Authors:** Marina Ramón-Gonçalves, Lorena Alcaraz, Susana Pérez-Ferreras, María Eugenia León-González, Noelia Rosales-Conrado, Félix A. López

**Affiliations:** 1 National Center for Metallurgical Research (CENIM), Spanish National Research Council (CSIC), Avda. Gregorio del Amo, 8, 28040 Madrid, Spain; 2 Institute of Catalysis and Petrochemistry (ICP), Spanish National Research Council (CSIC), C/Marie Curie, 2, 28049 Madrid, Spain; 3 Department of Analytic Chemistry, Faculty of Chemistry, Complutense University of Madrid (UCM), Avda. Complutense s/n, 28040 Madrid, Spain

**Keywords:** Environmental sciences, Solid Earth sciences

## Abstract

A valorization process of spent coffee grounds (SCG) was studied. Thus, a two-stage process, the first stage of polyphenols extraction and synthesis of a carbonaceous precursor and a subsequent stage of obtaining activated carbon (AC) by means of a carbonization process from the precursor of the previous stage, was performed. The extraction was carried out with a hydro-alcoholic solution in a pressure reactor, modifying time, temperature and different mixtures EtOH:H_2_O. To optimize the polyphenols extraction, a two-level factorial experimental design with three replicates at the central point was used. The best results were obtained by using a temperature of 80 °C during 30 min with a mixture of EtOH:H_2_O 50:50 (v/v). Caffeine and chlorogenic acid were the most abundant compounds in the analysed extracts, ranging from 0.09 to 4.8 mg∙g^−1^ and 0.06 to 9.7 mg∙g^−1^, respectively. Similarly, an experimental design was realized in order to analyze the influence of different variables in the AC obtained process (reaction time, temperature and KOH:precursor ratio). The best results were 1 h, 850 °C, and a mixture of 2.5:1. The obtained activated carbons exhibit a great specific surface (between 1600 m^2^∙g^−1^ and 2330 m^2^∙g^−1^) with a microporous surface. Finally, the adsorption capacity of the activated carbons was evaluated by methylene blue adsorption.

## Introduction

Coffee is a popular and consumed beverage worldwide and during the last 150 years has grown steadily in commercial importance^1,2^. Statistical evaluation reveals that around 50% of the coffee produced worldwide is used for drinking purposes^3^. As reported by the International Coffee Organization (ICO, 2018), 9.4 million tons of coffee were produced globally in 2018^4–7^, which entails a great generation of coffee waste. Worldwide, large amounts of coffee waste such as pulp, husk, coffee beans and spent coffee grounds are generated daily^7,8^. Around 650 kg of spent coffee grounds are generated by 1000 kg of green coffee beans processed^6^. In the upcoming years, with the high coffee production and the great number of waste generated^9^, it is necessary to find novel applications for reusing coffee residues, including those produced during coffee beverage preparation. Waste recycling offers many environmental, social and financial benefits^8,10,11^. The generated spent coffee grounds can be converted to biofuels, food additives, biosorbents, activated carbons, phenolic antioxidants, among other^1,3,10,12^. These residues contain substantial amounts of high value-added products such as carbohydrates, proteins, pectins and bioactive compounds like polyphenols^6^.

The polyphenols present in SCG are a group of secondary metabolites of plants, which are the constituents of a great number of fruit and vegetables, and beverages such as tea, coffee and wine and the main antioxidants in the human diet^13^. Thus, caffeic acid is a well-known non-flavonoid phenolic compound abundant in coffee, presenting potent antioxidant and neuroprotective properties^14^. Polyphenols might have different properties as an antioxidant, antiproliferative, anti-allergic, anticarcinogen, antimicrobial, antitumor, anti-inflammatory and neuroprotective activities^5,15^, that are of potential attention for the agrifood, cosmetic and pharmaceutical industries^6,16,17^. In this sense, several studies focused on the extraction of bioactive polyphenols from different food by-products such as agricultural residues, underutilized fruits, spent coffee ground (SCG)^18^, residual brewing yeast^19^ or beer residues^20^, citrus peels waste^21^, grape pomace seeds and skin^22^ or grapefruit solid waste have been described.

On the other hand, activated carbon (AC) is a porous solid that is used in many industrial sectors. Within its wide variety of applications, the most common use of AC is to removal several pollutants from wastewater due to its low cost and simplicity the process^9,23–25^. Their textural properties, including their high specific surface and microporosity, make it an ideal compound to remove contaminants through physical adsorption processes^26^. Other AC applications are the employment as a decolourizer for food industry, catalysis, purification steps for the chemical and pharmaceutical industry, for the elimination of gases, improving the noble metals or storing energy^27,28^. Commercial ACs are mostly obtained from biomass waste^29,30^, which after a carbonization process are used as a precursor of activated carbon such as residues derives from tea, coffee or grapes and olives bones^31–33^. One of the most used mechanisms for the synthesis of a carbonaceous precursor, which is used in this work, is hydrothermal carbonization. This mechanism is suitable for residues with high moisture content (>50% weight) -such as spent coffee grounds- obviating the need for energy drying before or during the process^34^. In addition, after the process, a liquid is obtained that contains compounds of value-added such as polyphenols and the use of a relatively low temperature (80 °C with the typical processing temperatures ranges for biomass from 270 °C to 370 °C)^35^ and low pressure (11 MPa), avoids degradation of the phenolic compounds present in the sample^36^.

There are previous investigations about the polyphenols extraction and the activated carbons obtention from coffee wastes. However, to the best of our knowledge, a process which involves both the polyphenols extraction and the subsequently activated carbon obtention have not been described. The aim of the present work was the development of a simple, easy and ecofriendly methodology in optimal experimental conditions that allow not only the synthesis of a suitable carbonaceous precursor used to obtain an activated carbon with high specific surface area by means of hydrothermal synthesis followed by a KOH chemical activation, based on the reuse of spent coffee grounds obtained after coffee beverage preparation, but also the recovery of an aqueous solution, from the hydrothermal synthesis, rich in polyphenols that can be identified and quantified.

## Materials and Methods

### Obtaining of the spent coffee ground (SCG) extracts

The waste recovered after coffee beverage preparation (SCG, spent coffee ground,) comes from the canteen of the National Center for Metallurgical Research (Superior Council of Scientific Investigations) in Madrid. The coffee ground used was a mixture of 10 (wt, %) torrefacto roasted and 90 (wt, %) natural roasted. Torrefacto coffee is a special class, highly consumed in Spain, obtained through coffee beans roasting in the presence of sugar to increase its flavour. Spent coffee grounds were generated after espresso extraction from the purchased coffee.

The SCG samples were maintained at −20 °C until analysis. Moisture content of the SCG was obtained by the sample drying at 80 °C during 48 h.

### Capillary liquid chromatography with ultraviolet detection (cLC-DAD) and spectrophotometric analysis

The instruments used to identify the different analytes were provided by the analytical department of the Complutense University of Madrid. These instruments were used in other similar analyzes^18,19^. An Agilent cLC Instrument Mod. 1100 Series (Agilent Technologies, Madrid, Spain) formed by a G1379A degasser, a G1376A binary capillary pump and a G1315B diode array detector (500 nL, 10 mm pathlength) was used for cLC analysis. A stainless steel loop with a volume of 10 µL was coupled to a Rheodyne injection valve. The capillary analytical column was a Synergy Fusion 4 µm C18 (150 mm × 0.3 mm I.D.) from Phenomenex (Torrance, CA, USA). Data acquisition and processing were performed with the Agilent Chemstation Software Package for Microsoft Windows.

Caffeine and polyphenols identification was carried out using a previously reported method by León-González *et al*.^19^. Wavelengths of 220, 260, 292, 310 and 365 nm were chosen for the UV-diode array detection. Quantitative analysis was realized at 260 nm for 3,4-dihydroxybenzoic, caffeine, rutin and quercetin, 292 nm for both naringin and gallic acid, 310 nm for chlorogenic acid, *trans*-ferulic acid resveratrol and *p*-coumaric acid and 365 nm for kaempferol.

Vijayalaxmi *et al*.^12^ and Shrikanta *et al*.^37^ modified spectrophotometric methods were employed for determining Total Flavonoid Content (TFC), Total Polyphenol Content (TPC) and Total Antioxidant Activity (TAA).

### Obtention and optimization of polyphenols extraction and activated carbon conditions by experimental design

The extraction of polyphenols from SCG samples were done in a Berghof BR3000 reactor at controlled temperature and pressure. An amount of 45 g of SCG were added to 600 mL of a hydro-alcoholic solution with different EtOH:H_2_O ratios (Table S1). The extraction time and the extraction temperature was modified between the range 15 and 30 min and 80–120 °C, respectively, while the pressure was kept constant at 50 bar. After cooling down at room temperature, the resulting suspension was centrifuged for 1 h. The solid obtained (H-SCG) was separated, and an aliquot of the resulting solution was used to quantify the individual polyphenol and caffeine present in the sample by cLC-DAD.

In addition, two-level factorial design with three replicates at the central point was planned to maximize the extracted amount of the target polyphenols and caffeine from SCG samples. For it, a chromatographic study for the identification of the experimental factors that could most influence in the polyphenol extraction (i.e. extraction time, extraction temperature, pressure and nature of the extraction solution) was carried out previously. As a result, nature of the extraction solution, extraction time and extraction temperature were selected as critical experimental factors and included in the experimental design. Extracted amount (mg∙g^−1^) of caffeic acid, rutin, *trans*-ferulic acid, naringin, kaempferol, resveratrol and caffeine were fixed as responses, and the optimization criterion for the analysis of the performed experimental design was the maximization of response values for the analytes evaluated. Table S1 summarized the different conditions used in the optimization process.

After the extraction process optimization, the hydro-alcoholic solution obtained under optimum conditions which containing the maximum amounts of the polyphenols extracted (WS), was evaporated in a rotavapor R-100 (Buchi) at 110 mbar (11 MPa) pressure and at a temperature of 40 °C, yielding a concentrated aqueous solution of polyphenols (CWS) and a fraction of ethyl alcohol, which could be reused in the extraction process.

On the other hand, the precursor obtained under optimum extraction conditions (experiment N°6, Table S1) was used to obtain AC by a method of chemical activation with KOH (Table S2). Thus, 1 g of the carbonaceous precursor was mixed with different amounts of KOH, between 1.5 and 2.5 g. The resulting mixtures were homogenized with a ball mill, placed in alumina crucibles and treated in a Carbolite STF 15 tubular furnace at 850 °C for different times under a nitrogen carrier (150 mL∙min^−1^). Once cooling to room temperature, the solid was washed with Milli-Q water until neutral pH. Then, it was dried in an oven at 80 °C during 12 h.

Activation degree (burn-off) and the yield of the activation were calculated from Eqs. 1 and 2:

1 $${\rm{Burn}}-{\rm{off}}( \% )=\frac{{{\rm{w}}}_{1}-{{\rm{w}}}_{2}}{{{\rm{w}}}_{1}}\cdot 100$$

2 $${\rm{Yield}}({\rm{wt}},\, \% )=\frac{{{\rm{w}}}_{2}}{{{\rm{w}}}_{1}}\cdot 100$$

where w_1_ and w_2_ are the mass (dry ash-free [daf] basis) of carbonaceous material before and after activation.

**Figure 1:**
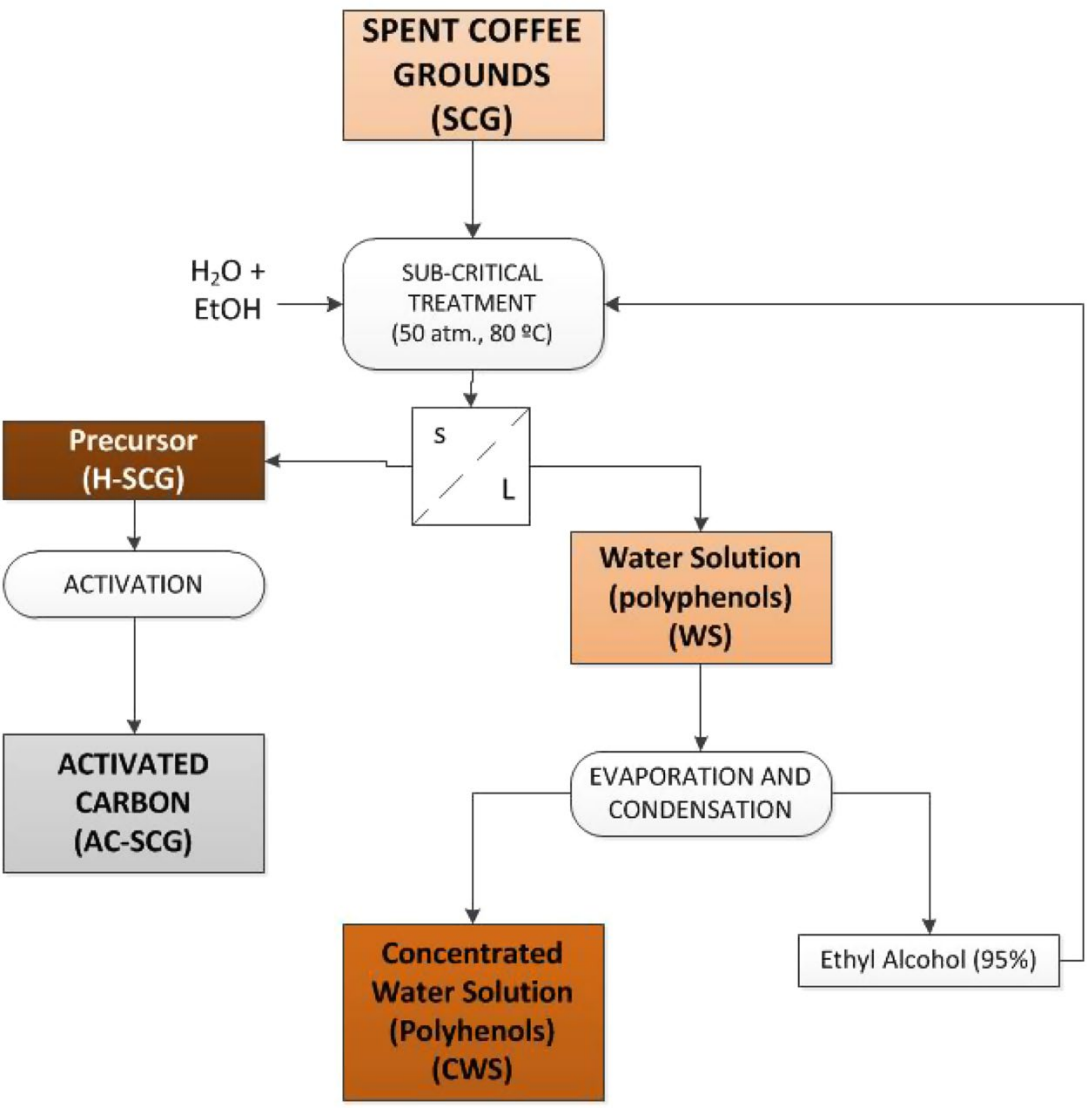
Diagram of the process studied.

In summary, spent coffee grounds were initially subjected to a hydro-alcoholic extraction process under subcritical conditions to obtain a liquid extract which contains the bioactive compounds and a precursor solid. Then, the liquid extract was evaporated at low pressure, allowing to recover the alcohol fraction (that could reuse it in the extraction stage) and an aqueous solution, in which the polyphenols were concentrated. Finally, the solid precursor was turned into activated carbons. Figure 1 schematically describes the described process.

### Characterization of the activated carbons

The porous structure of the AC was determined by nitrogen adsorption at −196 °C (77 K) using the Micromeritics ASAP 2020. The samples were partially degassed at 350 °C (623 K) for 16 h. The specific surface area was computed using the adsorption isotherm via the BET equation and DFT models, using Micromeritics and Quantachrome software. The surface of ACs was examined by field emission scanning electron microscope (FE-SEM) using a Hitachi S 4800 J microscope.

The textural properties of the obtained AC were optimized employing a two-level factorial design with three replicates at the centre point. Time, temperature and different amounts of KOH were selected as experimental factors. The total volume of pores (V_p_), volume of micropores (W_o_), the size of the micropores (L_o_), the microporous surface (S_mi_), the non-microporous external surface (S_e_) and the specific surface area (S_BET_) were chosen as responses, and the optimization criterion for the analysis of experimental design was maximization of the response values. Table S2 summarized the different conditions used in the optimization process.

### Batch adsorption experiments

The adsorption capacity of MB by the obtained AC was investigated. Different adsorption experiments were carried out. For it, 10 mg of the PCF-28 CA were added to MB solutions of concentration 10 mg∙L^−1^. The mixtures were magnetically stirred at 350 rpm in a thermostatic-controlled bath. Aliquots were extracted every 5 min (up to 30 min), every 10 min (up to 60 min) and finally, every 60 min (until equilibrium is reached). The amount of the MB in solution was calculated by UV-Vis spectroscopy at 610 nm employing a Zuzi Spectrophotometer 4101.

The adsorbed MB amount per gram of AC (q_e_) was calculated from Eq. 3:

3 $${{\rm{q}}}_{{\rm{e}}}=\frac{({{\rm{C}}}_{0}-{{\rm{C}}}_{{\rm{e}}}){\rm{V}}}{{\rm{m}}}$$

where C_o_ and C_e_ are the initial and at the equilibrium MB concentration in the solution (mg∙L^−1^), respectively, V is the volume of the solution (L) and m is the mass of the AC (g) employed.

In order to evaluate the thermodynamic study, the linear form of the Langmuir^38^ (Eq. 4), Freundlich^39^ (Eq. 5) and Temkin^40^ (Eq. 6) isotherms were employed.

4 $$\frac{{{\rm{C}}}_{{\rm{e}}}}{{{\rm{q}}}_{{\rm{e}}}}=\frac{1}{{{\rm{q}}}_{{\rm{m}}}{\rm{b}}}+\frac{1}{{{\rm{q}}}_{{\rm{m}}}}{{\rm{c}}}_{{\rm{e}}}$$

5 $$\mathrm{ln}\,{{\rm{q}}}_{{\rm{e}}}=\,\mathrm{ln}\,{{\rm{K}}}_{{\rm{F}}}+\frac{1}{{\rm{n}}}\,\mathrm{ln}\,{{\rm{c}}}_{{\rm{e}}}$$

6 $${{\rm{q}}}_{{\rm{e}}}=B\cdot \,\mathrm{ln}\,{{\rm{A}}}_{{\rm{T}}}+\frac{RT}{{b}_{T}}\cdot \,\mathrm{ln}\,{{\rm{c}}}_{{\rm{e}}}$$

where C_e_ and q_e_ are the MB concentration (mg∙L^−1^) and the MB amount adsorbed per mass of AC at equilibrium (mg∙g^−1^), respectively; q_m_ is the maximum adsorption capacity of the AC (mg∙g^−1^) and b is the Langmuir equilibrium constant (L∙mg^−1^); K_F_ (L∙g^−1^) and n are adsorption constants; A_T_ is the Temkin isotherm equilibrium binding constant (L∙g^−1^), b_T_ is the Temkin isotherm constant and R is the universal gas constant (8.314·10^3^ kJ∙K^−1^∙mol^−1^).

Moreover, non-dimensional Langmuir constant^41^ (R_L_) was determined using Eq. 7:

7 $${{\rm{R}}}_{{\rm{L}}}=\frac{1}{1+{{\rm{bC}}}_{0}}$$

where C_0_ is the initial concentration of MB (mg∙L^−1^).

R_L_ value could indicate that the adsorption process is unfavourable (R_L_ > 1), linear (R_L_ = 1), favourable (0 < R_L_ < 1) or irreversible (R_L_ = 0)^42^.

The kinetic data were also analyzed using a pseudo-first-order^43^ (Eq. 8) and pseudo-second-order models^44^ (Eq. 9):

8 $$\mathrm{ln}({{\rm{q}}}_{{\rm{e}}}-{{\rm{q}}}_{{\rm{t}}})=\,\mathrm{ln}\,{{\rm{q}}}_{{\rm{e}}}-{{\rm{K}}}_{1}\cdot {\rm{t}}$$

9 $$\frac{{\rm{t}}}{{{\rm{q}}}_{{\rm{t}}}}=\frac{1}{{{\rm{K}}}_{2}{{\rm{q}}}_{{\rm{e}}}^{2}}+\frac{1}{{{\rm{q}}}_{{\rm{e}}}}\cdot {\rm{t}}$$

where K_1_ and K_2_ are the pseudo-first (min^−1^) and pseudo-second-order (g∙min∙mg^−1^) adsorption constants.

Finally, the thermodynamic equilibrium constant as the Gibbs free energy (ΔG^0^) (Eq. 10), the standard enthalpy (ΔH^0^) and the entropy (ΔS^0^) (Eq. 11) were determined using the following equations^45^:

10 $${{\rm{\Delta }}G}^{0}=-\,{\rm{RT}}\,\mathrm{ln}\,{{\rm{K}}}_{{\rm{\alpha }}}$$

11 $$\mathrm{ln}\,{{\rm{K}}}_{{\rm{\alpha }}}=\frac{{{\rm{\Delta }}{\rm{S}}}^{0}}{{\rm{R}}}-\frac{{{\rm{\Delta }}{\rm{H}}}^{0}}{{\rm{RT}}}$$

where K_α_ is the thermodynamic equilibrium constant (Eq. 12):

12 $${{\rm{K}}}_{{\rm{\alpha }}}=\frac{{{\rm{C}}}_{{\rm{s}}}}{{{\rm{C}}}_{{\rm{e}}}}$$

where C_s_ and C_e_ are the solid (mg∙g^−1^) and liquid (mg∙L^−1^) phase concentration at equilibrium.

## Results and Discussion

### Optimization of polyphenol extraction conditions by experimental design

For optimization of the polyphenol extraction conditions from spent coffee ground (SCG), a two-level factorial experimental design was planned. According to Table S1, a total of 11 runs were performed. The obtained extracts for each tested condition were analyzed by capillary LC-DAD allowing both the individual detection and quantification of extracted polyphenols. Analysis of the experimental results allowed determining the best experimental extraction conditions for achieving the maximum response with regard to the composition of the extraction solvent (EtOH:H_2_O mixture), temperature and extraction time. The extracted amounts for each detected polyphenol from the 11 tested conditions are shown in Table S3. Among them, n° 6 and n° 8 experiments (Table S3) exhibit the highest polyphenols content. Experiment n° 6 showed the highest extracted amounts (mg∙g^−1^) for caffeine, caffeic acid and *trans*-ferulic acid, while for rutin and naringin the conditions of experiment n° 8 provided higher contents. For resveratrol, a similar concentration was obtained in all the experiments, whereas in the case of kaempferol, its concentration was below the limit of quantification (LOQ) determined by cLC-DAD. In order to determine the significant effects and the interactions between them, an estimated normalized response surfaces for each analyte were plotted. As an example, Fig. S1 shows the three-dimensional graphs obtained for caffeic acid and rutin in the extracts obtained from spent coffee ground sample (SCG) under specific extraction conditions. The caffeic acid amount increased with the EtOH:H_2_O ratio and the extraction time, while the extraction time and temperature have more influence in the rutin concentration. So, the obtained results indicate that it is not possible to obtain the maximum amount of the analytes in the same extraction conditions. For this reason, a multiple response analysis (MRA) was carried out. In this way, it is possible to determine the combination of the experimental factors which simultaneously optimized the studied responses. As a compromise, the optimum conditions to obtain the maximum responses for the target analytes were 30 min for the extraction time, a temperature of 80 °C, and a mixture of EtOH:H_2_O (50:50, v/v) as extraction solvent. Under these conditions, experimental responses were in agreement with those predicted by means of the experimental design analysis.

### Polyphenols determination by cLC-DAD in the extracts from SCG

**Figure 2:**
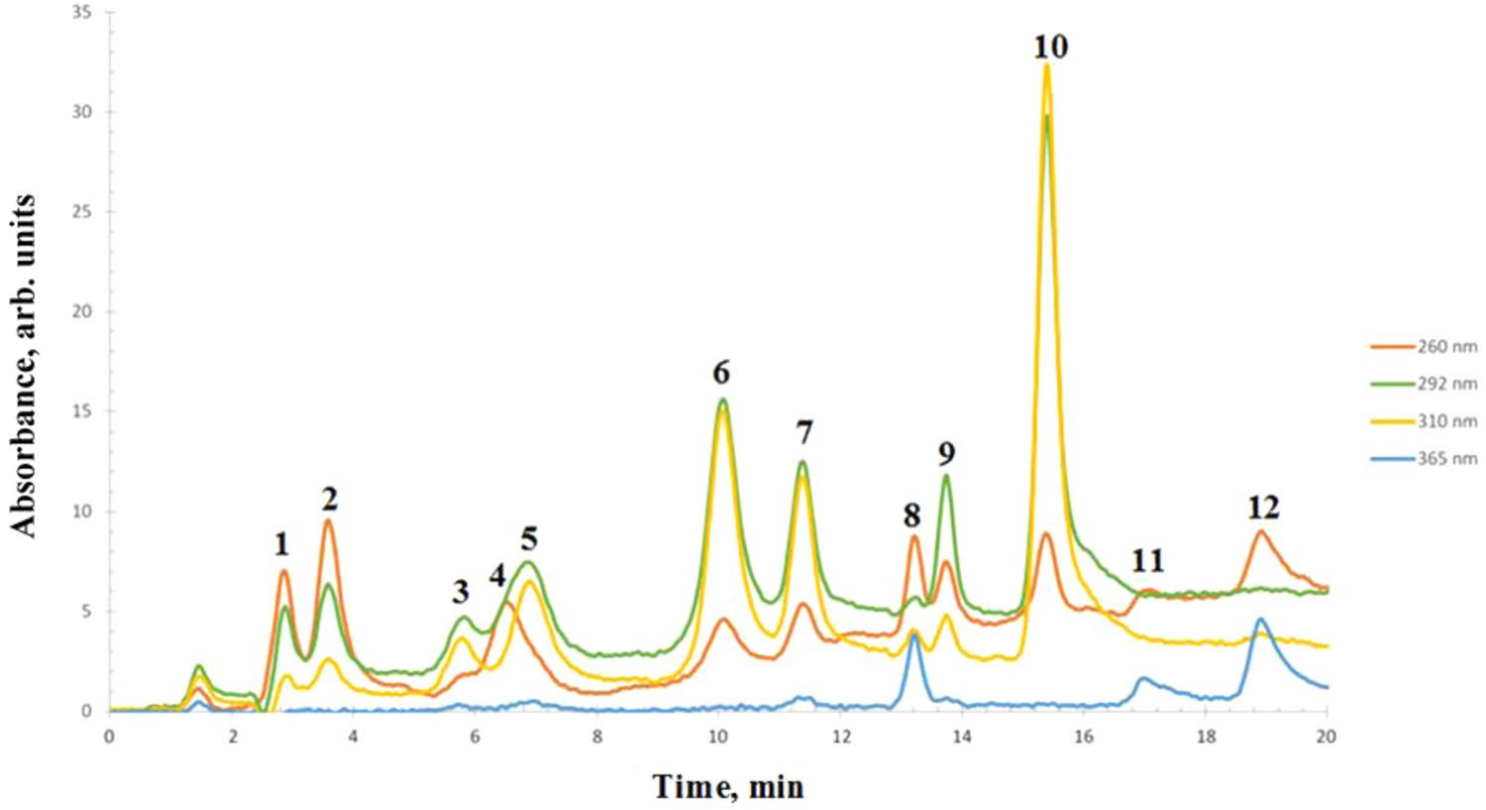
Chromatogram of a standard mixture (80 µg/L) obtained by the cLC-DAD optimized method. (1) Gallic acid; (2) Dihydroxybenzoic acid; (3) Chlorogenic acid; (4) Caffeine; (5) Caffeic acid; (6) p-Coumaric acid; (7) Trans- Ferulic acid; (8) Rutin; (9) Naringin; (10) Resveratrol; (11) Quercetin; (12) Kaempferol.

Individual polyphenols determination before and after the evaporation process (CWS and organic solution) were carried out by capillary liquid chromatography (cLC-DAD). Figure 2 shows the chromatogram registered for a mixture of the standards was used for the identification of the different polyphenols studied and caffeine. In addition, Table 1 shows the extracted amount (mg analyte·g^−1^ dried sample) in the different evaluated extracts. As can be observed, CWS exhibit the highest polyphenols and caffeine (except rutin and kaempferol) content followed by SCG, while in the other extracts (WS, H-SCG and ethanol) lower amounts were found. Furthermore, it is observed that the evaporation process leads to concentrate polyphenol and caffeine compounds in the aqueous solution, while residual amounts remain in the organic solution.

**Table 1:** Amounts of phenolic compounds and caffeine extracted from different solute.

Compound (mg·g^−1^)*	Sample Code				
	SCG	H-SCG	WS	CWS	Ethanol
Caffeine	2.476	0.26	0.99	4.833	0.089
Chlorogenic acid	LOD	LOD	n.d.	9.689	LOD
*p*-Coumaric acid	0.155	0.229	n.d.	0.348	0.046
*trans*- Ferulic acid	0.166	0.157	0.110	0.177	0.024
Rutin	0.086	LOD	0.064	LOD	LOD
Naringin	0.214	0.096	0.143	0.423	0.010
Resveratrol	0.071	0.066	0.07	0.093	0.013
Quercetin	0.076	0.073	n.d.	0.095	0.015
Kaempferol	0.053	0.098	LOD	0.028	0.014

*Extracted amounts are expressed as mg per gram of dried sample. LOD: determined at the concentration levels of the method detection limit, n.d: no detected, SCG = extract from spent coffee grounds, H-SCG = extract from precursor, WS = extract obtained before the evaporation process, CWS = concentrated extract obtained after the evaporation process, Ethanol = organic solution obtained after the evaporation process.

### Estimation of TPC, TFC, and TAA in the extracts obtained from SCG samples

Total polyphenol (TPC), flavonoid content (TFC), and antioxidant activity (TAA) indexes of the obtained extracts from SCG under the optimum extraction conditions mentioned above were experimentally evaluated. The results obtained by applying spectrophotometric methods for the extract before the evaporation (i.e. WS) were (14 ± 8) mg GAE∙g^−1^ DW, (10.7 ± 0.8) mg QE·g^−1^ DW, (14.5 ± 0.3) mg GAE∙g^−1^ DW, for TPC, TFC and TAA, respectively. In addition, in the case of the CWS (the obtained extract after evaporation process), (49 ± 1) mg GAE∙g^−1^ DW, (56 ± 7) mg QE·g^−1^ DW, (23 ± 2) mg GAE∙g^−1^ DW for TPC, TFC and TAA were obtained. Results are expressed per gram of dried (DW) sample and they are related to the amount of gallic acid (GAE) or quercetin (QE). As can be observed, after the evaporation process (WS extract), there was an increase in the TFC and TPC and TAA estimated values.

### Optimization of textural and morphological properties by factorial experimental design

For optimization of the textural and morphological properties of AC from precursor (H-SCG) under the optimum extraction conditions, a factorial experimental design was planned again (Table S2). Once a total of 11 experiments were performed. The subsequent analysis of the experimental results allowed to determine the best conditions for the maximum response. Table S4 shows the textural properties of the ACs obtained in the different experiments analyzed. The obtained results for S_mi_, S_e_ and S_BET_ showed significant effects for both time and temperature factors, and for interaction factors between temperature and KOH:precursor. As can be observed, the ACs have a microporous microstructure (S_mi_ ≈ S_BET_), their volume of micropores (W_o_) is similar to the pore volume (V_p_). The pore sizes (L_o_) are, in all cases, less than 2.2 nm. The BET surface area varies between 1377–2330 m^2^∙g^−1^. In general, an increase in the values of the variables studied can be observed in those experiments that have the highest temperature (850 °C). The nitrogen adsorption isotherms carried out at −196 °C for ACs of the experiments n°3 and n°5, that correspond to those that have the highest and lowest specific surface of all the active carbons obtained, are shown in Fig. S2. According to the International Union of Pure and Applied Chemistry (IUPAC), the shapes of these isotherms are of type I for experiment n° 5 and II for the experiment n° 3. At low relative pressures (p/p_0_ < 0.1), large volumes of N_2_ were adsorbed for both ACs samples^46^, which is typical of microporous solids The quantity adsorbed of N_2_ at p/p_0_ ~ 1 range between 350 and 758 cm^3^∙g^−1^.

Similar to the polyphenol extraction studies, estimated normalized response surfaces were plotted for the studied responses. Figure S3 shows the three-dimensional graphs obtained for S_BET_ and W_o_ parameters in the sample studied. The rest of response presented similar surfaces to those shown in Fig. S3, where the response variable increases with the temperature, which corroborates the results previously mentioned. Moreover, it was necessary again to carry out MRA due to the heterogeneity in the experimental conditions. As a compromise, the optimum conditions to obtain the maximum responses were a time of 1 h, a temperature of 850 °C, and a mixture of KOH:precursor at the ratio 2.5:1.

### Characterization of the active carbons

Regarding the characterization of active carbon, Table 2 exhibits burn-off and the yield of the activation process in the experimental assays carried out by means of the experimental design. As can be observed, when the carbonization temperature increases, the burn-off does it and the AC recovery (wt %) decreases. The burn-off values range between 82% and 96% and the yields between 4% and 18.5%. A comparative of the initial residue (SCG), the precursor (H-SCG) and optimized activated carbon (AC-SCG-8) elemental chemical compositions are shown in Table S5. Chemical analysis showed a high carbon content for SCG and H-SCG (51.2% and 51.6%, respectively) and as consequence of the carbonization process used the C content of the AC-SCG-8 was increased up to 84% in the activated carbon compared to the initial precursor. In addition, Table S5 also showed a low sulphur content (0.1%) for the three samples analyzed.

**Table 2:** The variation of the burn-off and yield of the activation process.

Experiment	Burn-off (wt, % daf)	Yield (wt, %)
AC-SCG-1	82	18
AC-SCG-2	82	18
AC-SCG-3	95	5
AC-SCG-4	96	4
AC-SCG-5	81	18
AC-SCG-6	86	13
AC-SCG-7	93	7
AC-SCG-8	96	4
AC-SCG-9	88	12

**Figure 3:**
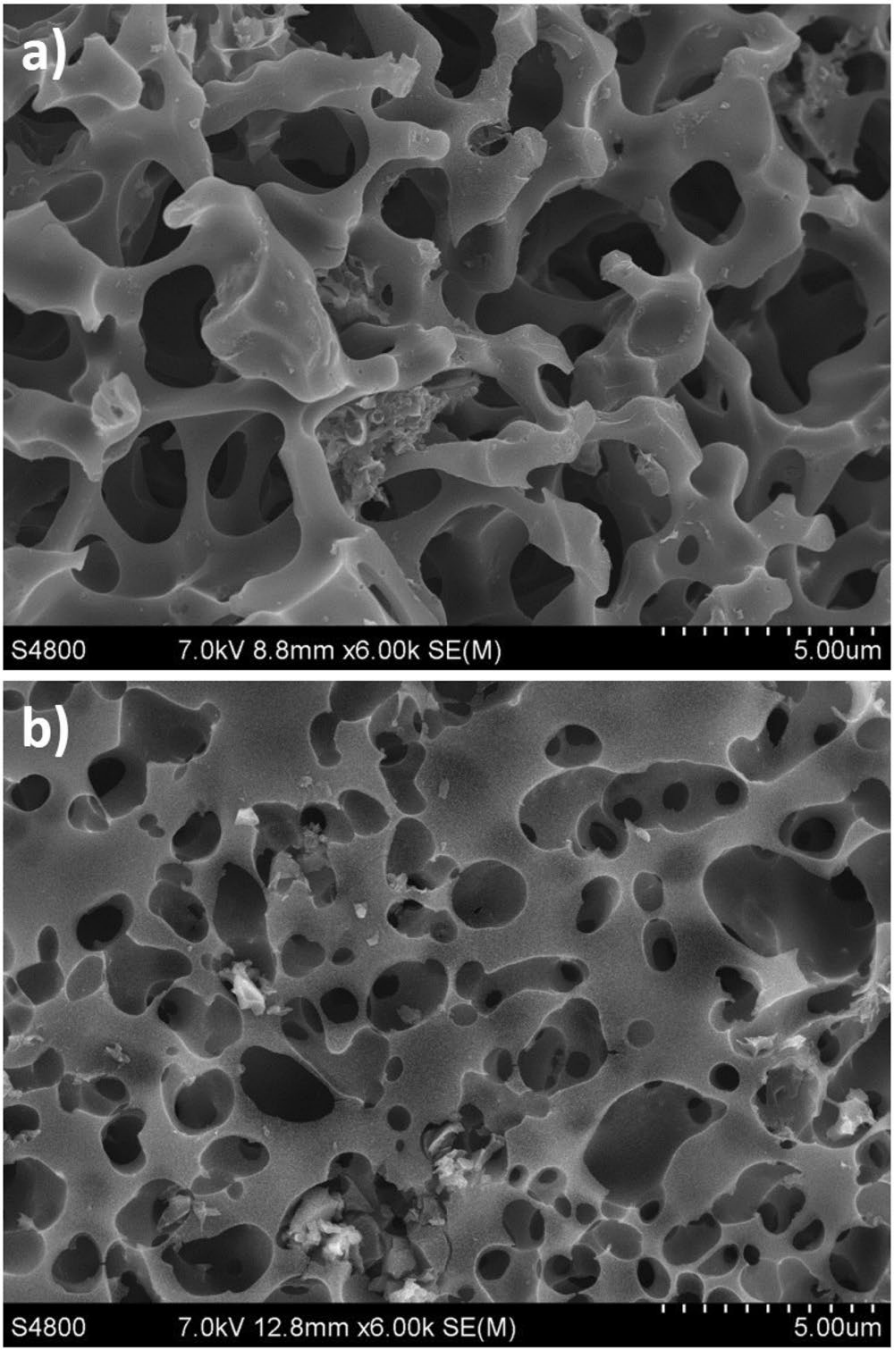
Morphology of activated carbons (ACs) obtained from experiment 5 (**a**) and experiment 3 (**b**) examined by FE-SEM.

Figure 3 shows, as an example, FE-SEM micrographs of the ACs obtained from the n° 5 (a) and n° 3 (b) experiments. These FE-SEM images were taken in order to observe the surface morphology of the samples. The images of the ACs show a lot of cavities indicating a microporous nature in both samples, according to the results obtained in the characterization of the ACs obtained. However, the AC obtained from the n° 3 experiment exhibits a greater number of pores compared with the AC obtained under the conditions of experiment n° 5.

### Adsorption of methylene blue

#### Thermodynamic adsorption studies

In order to analyze the possible MB adsorption mechanism, different isotherms were assessed at different temperatures. The calculated parameters values for the different isotherms in the linear form, using the Eqs. 2–4, are summarized in Table 3. The highest obtained R^2^ correlation coefficient values were for the Langmuir isotherm.

**Table 3:** Langmuir, Freundlich and Temkin calculated parameters for MB adsorption.

T (°C)	Langmuir				Freundlich				Temkin		
	q_m_ (mg∙g^−1^)	B (mg^−1^)	R_L_	R^2^	K_f_ (g^−1^)	n	1/n	R^2^	A_T_	b_T_	R^2^
30	411.5	18.7	0.003	0.999	340.8	6.4	0.2	0.972	4011.9	60.3	0.962
60	775.2	8.9	0.006	0.999	567.2	3.6	0.3	0.819	224.5	24.9	0.921
80	813.1	3.7	0.013	0.993	510.9	3.9	0.3	0.988	400.3	31.5	0.905

The calculated values q_m_ increase with the temperature, in accordance with previous studies related to the MB adsorption by ACs obtained from different wastes^32,47^. In addition, the calculated values are similar to the q_m_ experimental obtained (between 411–813 mg∙g^−1^).

The calculated Langmuir non-dimensional factor (R_L_) in all cases were between the range of 0 < R_L_ < 1. These results reveal that the methylene blue adsorption by the obtained activated carbon is a favourable process^41^ independently of the temperature used.

Thermodynamic parameters were calculated at different temperatures using Eqs. 10 and 11. The negative value obtained of enthalpy (∆H°) (−77.81 J·mol^−1^) indicates that the process is exothermic. In addition, the entropy (∆S°) was positive, 68.10 J·mol^−1^·K^−1^, which indicate the increased disorder at the solid-solution interface components. Finally, the free Gibbs energies (∆G°) (−205.73 kJ·mol^−1^ at 30 °C; −226.17 kJ·mol^−1^ at 60 °C and 239.79 kJ·mol^−1^ at 80 °C) were also negatives and exhibit that this process is spontaneous and favourable thermodynamically.

The obtained negative value of enthalpy (∆H°) indicates that the process is exothermic. In addition, the entropy (∆S°) was positive, which indicate the increased disorder at the solid-solution interface components. Finally, the free Gibbs energies (∆G°) were also negatives and exhibit that this process is spontaneous and favourable thermodynamically.

#### Kinetic adsorption studies

The adsorption kinetics experiments were also realized at different temperatures. Table 4 exhibits the obtained results. A pseudo-second-order model was the best fit in all cases, such as indicated the correlation coefficients obtained. K_2_ constant increased with temperature. The obtained results indicate that an increment of the temperature enhances the MB adsorption. The process activation energy (E_a_) was calculated fitting the observed kinetics rate constants logarithm (ln K_2,obs_) versus the inverse of temperature (1/T)^48^, where the slope is −E_a_/R. This value is used to estimate whether the process is a physical (readily reversible reactions and the energy requirements are small between the range of 5 to 40 kJ∙mol^−1^ or chemical adsorption (a process that requires higher energies, between 40 to 800 kJ∙mol^−1^, with stronger forces)^48^. The calculated activation energy in the present case was 16.25 kJ·mol^−1^. The obtained value could be suggested a physic-sorption process.

**Table 4:** Kinetic parameters and correlation coefficients for the MB adsorption.

T (°C)	Pseudo-first order			Pseudo-second order		
	k_1_ (min^−1^)	q_e_ (mg·g^−1^)	R^2^	k_2·_(10^−3^) (g∙min∙mg^−1^)	q_e_ (mg∙g^−1^)	R^2^
30	0.046	38.05	0.871	2.85	201.99	0.999
60	0.097	92.5	0.965	3.77	200.49	0.999
80	0.101	36.73	0.877	7.60	199.74	0.999

Isothermal and kinetic models, q_m_ values and activation agents of different adsorbents are exhibit in Table 5. As can be observed, many cases the KOH is used as an activation agent. In general, a better fit to the Langmuir model isotherm was found for the adsorption isotherms. Moreover, the adsorption kinetics were best fitted a pseudo-second-order model in the majority of the presented cases. With respect to the adsorption capacity, spent coffee grounds, wine wastes and wastes carpets have the highest adsorption capacity values (q_m_) while orange peel has the lowest value.

**Table 5:** Maximum monolayer adsorption capacity (q_m_), isothermal and kinetic models of various adsorbents for MB adsorption.

Precursor of the activated carbon	Activation agent	q_m_ (mg·g^−1^)	Isothermal model	Kinetic model	References
Spent coffee grounds^†^	KOH	411–813	Langmuir	Pseudo-second order	This work
Wine wastes^†^	KOH	714–847	Langmuir	Pseudo-second-order	(Alcaraz *et al*.)^32^
Wastes carpets	H_3_PO_4_	403–769.2	Langmuir	Pseudo-second-order	(Hassan *et al*.)^49^
Date stones	ZnCl_2_	398.2	Langmuir	Pseudo-second-order	(Ahmed *et al*.*et al*.)^50^
Bamboo	KOH	454.2	Langmuir	Pseudo-second-order	(Hameed *et al*.)^51^
Coconut husk	KOH	437.8	Langmuir	Pseudo-second-order	(Tan *et al*.)^52^
Vetiver roots	H_3_PO_4_	423	Langmuir	Pseudo-second-order	(Altenor *et al*.)^53^
Acorn shell	ZnCl_2_	312.5	Langmuir	—	(Altintig *et al*.)^27^
Sucrose^†^	KOH	704.2	Langmuir	Elovich	(Bedin *et al*.)^54^
Hazelnut shells^†^	KOH	524	Langmuir	Pseudo-second-order	(Unur *et al*.)^55^
Posidonia oceanica (L.)	ZnCl_2_	285.7	Langmuir	Dubinin-Raduchkevich	(Dural *et al*.)^56^
Orange peel	ZnCl_2_	150	—	—	(Köseoʇlu *et al*.)^57^
Peach Stones	H_3_PO_4_	444.3	Tóth and RedlichPeterson	—	(Álvarez-Torrellas *et al*.)^58^
Grape waste	ZnCl_2_	417	Langmuir	—	(Sayʇili *et al*.)^59^
Waste tea	CH_3_COOK	554.3	Langmuir	—	(Auta *et al*.)^60^

^†^Hydrothermal treatment for obtained the precursor.

## Conclusions

A valorization process to re-use spent coffee ground was assessed. A hydrothermal treatment was employed to obtain a hydro-alcoholic extraction solution of the inherent high added value polyphenols, in addition to a carbonaceous precursor used for a subsequent transformation into activated carbon (AC). Factorial experimental designs were used to optimize the polyphenols extraction conditions and the activated carbons obtention. For it, extraction time, extraction temperature and EtOH:H_2_O ratio were evaluated to the polyphenols extraction. 30 min of the extraction time, a temperature of 80 °C, and EtOH:H_2_O ratio (50:50, v/v) as extraction solvent were found to obtain the maximum responses. With respect to the activated carbon obtention, time and temperature of the calcination and KOH:precursor ratio have been evaluated. In this case, the optimum conditions to obtain the maximum responses were 1 h, 850 °C, and a mixture of KOH:precursor ratio 2.5:1. The obtained extracts are characterized by high values of total flavonoid content, in a range between 11–56 mg QE∙g^−1^ DW, and total polyphenol content, in a range between 14–49 mg GAE∙g^−1^ DW, as well as considerable antioxidant activities. In addition, all obtained activated carbons exhibit a microporous structure, with high specific surfaces, between 1600 m^2^∙g^−1^ and 2330 m^2^∙g^−1^, provides them with excellent adsorption properties. Methylene blue adsorption capacity onto the obtained activated carbon have been evaluated. Thermodynamic and kinetic studies were assessed. Langmuir isotherm and pseudo-second order model were the best fitted obtained. Finally, a thermodynamic study reveals that the MB adsorption is a spontaneous and favourable process. All these results yield high-quality AC and extracts with high value-added compounds, from spent coffee grounds, with potential interest for many industries. The present work also represents a promising alternative for reusing and valorization of this coffee residue, which is produced in very high amounts and it is commonly used as organic fertilizer.

## Supplementary information

Supplementary information
