# Supplementary material for: Extraction of polyphenols and synthesis of new activated carbon from spent coffee grounds
Source: Sci Rep. 2019 Nov 27;9:17706. doi: 10.1038/s41598-019-54205-y (PMC6881328; doi:10.1038/s41598-019-54205-y)
Supplement: Supplementary file 1 — Supplementary information [file 41598_2019_54205_MOESM1_ESM.docx]

**Extraction of polyphenols and synthesis of new activated carbon from spent coffee grounds**

Marina Ramón Gonçalves^1^, Lorena Alcaraz^1^, Susana Pérez-Ferreras^2^, María Eugenia León-González^3^, Noelia Rosales-Conrado^3^, Félix A. López^1,*^

^1^ National Center for Metallurgical Research (CENIM), Spanish National Research Council (CSIC), Avda. Gregorio del Amo, 8, 28040 Madrid, Spain

^2^ Institute of Catalysis and Petrochemistry (ICP). Spanish National Research Council (CSIC), C/ Marie Curie, 2. 28049 Madrid, Spain

^3^ Department of Analytic Chemistry, Faculty of Chemistry, Complutense University of Madrid (UCM), Avda. Complutense s/n, 28040 Madrid, Spain

^*^Corresponding Author: f.lopez@csic.es

**Supplementary Figures**

Figure S1.

**
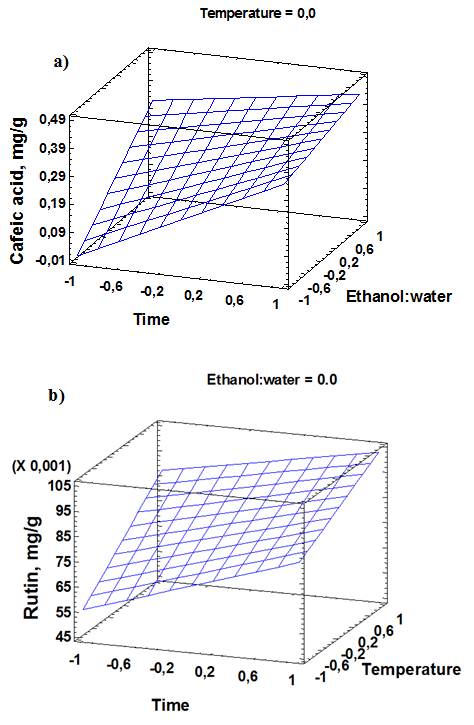
**

Figure S1. Estimated normalized response surfaces obtained for the content (mg∙g^-1^) of cafeic acid (a) and rutin (b) extracted from spent coffee ground sample (WS), both at different extraction times and ethanol:water mixtures or temperatures. Temperature was fixed at 100ºC (a), ethanol:water ratio at 35:65 (v/v) (b).

Figure S2.


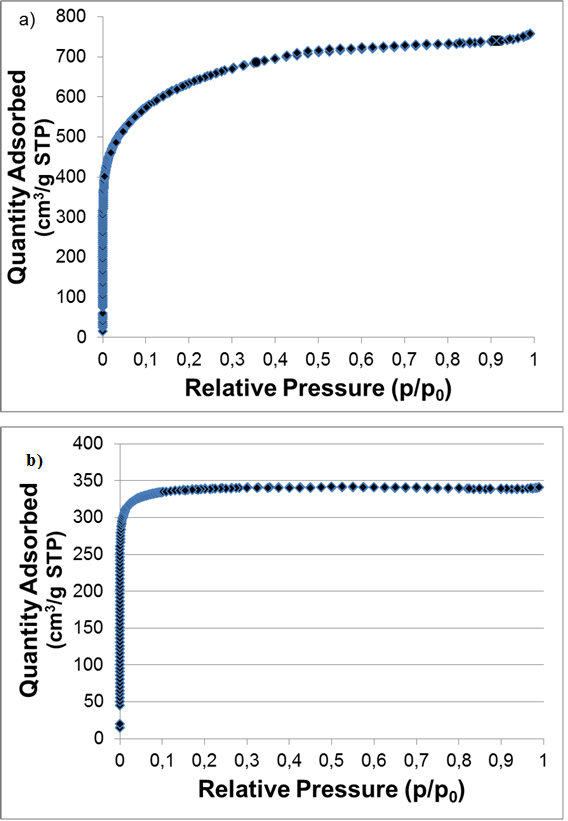


Figure S2. N_2_ adsorption isotherms at 77 K for the activated carbons of the experiments N^o^. 3 (a) and 5 (b).

Figure S3.


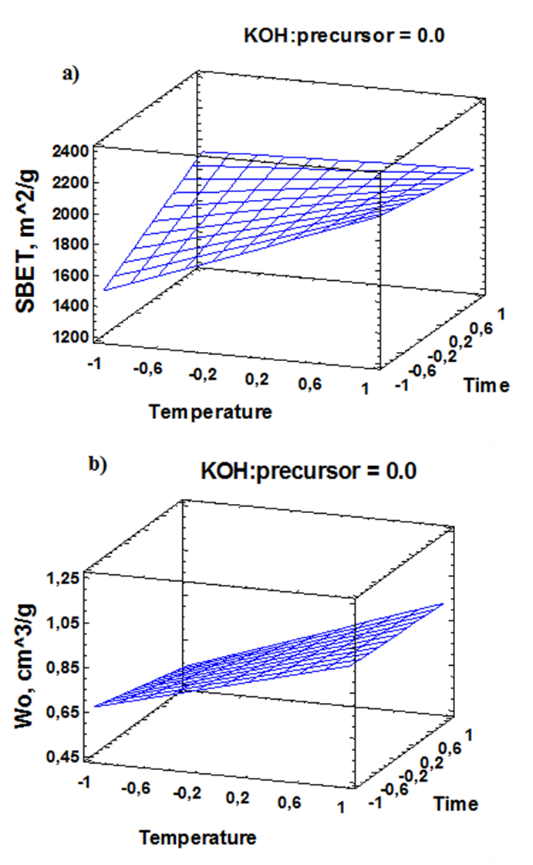


Figure S3. Estimated normalized response surfaces obtained for the SBET (a) and Wo (b) from optimized active carbon (AC-SCG), both at different times and temperatures. KOH:precursor was fixed at 2:1 (m/m).

**Supplementary table**

Table S1. Plan of experiments for the two-level factorial design aimed to optimization of polyphenol extraction conditions.

| **Experiment**  **Number** | **Factors** | | |
| --- | --- | --- | --- |
|  | **Extraction solvent**  **(EtOH:H_2_O, v/v)** | **Extraction time (min)** | **Temperature (ºC)** |
| 1 | 50:50 (1) | 30 (1) | 120 (1) |
| 2 | 50:50 (1) | 30 (1) | 80 (-1) |
| 3 | 50:50 (1) | 15 (-1) | 120 (1) |
| 4 | 50:50 (1) | 15 (-1) | 80 (-1) |
| 5 | 30:70 (-1) | 30 (1) | 120 (1) |
| 6 | 30:70 (-1) | 30 (1) | 80 (-1) |
| 7 | 30:70 (-1) | 15 (-1) | 120 (1) |
| 8 | 30:70 (-1) | 15 (-1) | 80 (-1) |
| 9 | 40:60 (0) | 23 (0) | 100 (0) |
| 10 | 40:60 (0) | 23 (0) | 100 (0) |
| 11 | 40:60 (0) | 23 (0) | 100 (0) |

Normalized factors are indicated in parenthesis.

Table S2. Plan of experiments for the two-level factorial design for obtaining activated carbon from the solid residue recovered after polyphenol extraction.

| **Experiment**  **Number** | **Factors** | | |
| --- | --- | --- | --- |
|  | **Time (min)** | **Temperature (ºC)** | **KOH:precursor** |
| 1 | 30 (-1) | 700 (-1) | 1.5:1 (-1) |
| 2 | 30 (-1) | 700 (-1) | 2.5:1 (1) |
| 3 | 30 (-1) | 850 (1) | 1.5:1 (-1) |
| 4 | 30 (-1) | 850 (1) | 2.5:1 (1) |
| 5 | 60 (1) | 700 (-1) | 1.5:1 (-1) |
| 6 | 60 (1) | 700 (-1) | 2.5:1 (1) |
| 7 | 60 (1) | 850 (1) | 1.5:1 (-1) |
| 8 | 60 (1) | 850 (1) | 2.5:1 (1) |
| 9 | 45 (0) | 775 (0) | 2:1 (0) |
| 10 | 45 (0) | 775 (0) | 2:1 (0) |
| 11 | 45 (0) | 775 (0) | 2:1 (0) |

Normalized factors are indicated in parenthesis.

Table S3. Phenolic compounds extracted from spent coffee grounds.

| **Compound**  **(mg∙g^-1^)^*^** | **Experimental design number** | | | | | | | | | | | |
| --- | --- | --- | --- | --- | --- | --- | --- | --- | --- | --- | --- | --- |
|  | **t_R_**  **(min)** | **1** | **2** | **3** | **4** | **5** | **6** | **7** | **8** | **9** | **10** | **11** |
| Caffeine | 7.2 | 0.420 | 0.469 | 0.333 | 0.450 | 0.449 | 1.090 | 0.890 | 1.008 | 0.966 | 1.241 | 0.963 |
| Cafeic acid | 7.7 | n.d | 0.354 | n.d | 0.299 | 0.337 | 0.469 | 0.344 | 0.396 | 0.429 | n.d | 0.439 |
| *trans*-  Ferulic acid | 12.2 | 0.071 | 0.067 | 0.042 | 0.047 | 0.074 | 0.121 | 0.084 | 0.088 | 0.095 | 0.073 | 0.097 |
| Rutin | 13.6 | 0.045 | 0.066 | 0.087 | 0.089 | 0.075 | 0.090 | 0.103 | 0.104 | 0.082 | 0.065 | 0.085 |
| Naringin | 14.4 | 0.096 | 0.127 | 0.119 | 0.145 | 0.110 | 0.107 | 0.138 | 0.142 | 0.092 | 0.089 | 0.103 |
| Resveratrol | 16.2 | 0.075 | 0.075 | 0.077 | 0.092 | 0.081 | 0.089 | 0.083 | 0.097 | 0.057 | 0.044 | 0.058 |
| Kaempferol | 19.7 | LOD | LOD | LOD | 0.004 | LOD | 0.004 | LOD | LOD | LOD | LOD | LOD |

^*^ Extracted amounts are expressed as mg per gram of dried sample. LOD: determined at the levels of the method detection limit, n.d: no detected.

Table S4. Porosity features of the activated carbons

| **Experiment**  **number** | **V_p_**  **(cm^3^∙g)** | **Wo**  **(cm^3^∙g)** | **Lo**  **(nm)** | **S_mi_**  **(m^2^∙g)** | **S_e_**  **(m^2^∙g)** | **SBET**  **(m^2^∙g)** |
| --- | --- | --- | --- | --- | --- | --- |
| AC-SCG-1 | 0.675 | 0.620 | 1.680 | 1570.451 | 37.481 | 1607.9 |
| AC-SCG-2 | 0.826 | 0.715 | 1.759 | 1814.409 | 63.092 | 1877.5 |
| AC-SCG-3 | 1.168 | 1.067 | 2.005 | 2265.062 | 65.591 | 2330.6 |
| AC-SCG-4 | 0.992 | 0.856 | 2.115 | 1789.504 | 85.874 | 1867.3 |
| AC-SCG-5 | 0.527 | 0.509 | 1.531 | 1346.483 | 30.533 | 1377.0 |
| AC-SCG-6 | 0.609 | 0.585 | 1.582 | 1516.526 | 22.780 | 1539.3 |
| AC-SCG-7 | 0.911 | 0.837 | 1.894 | 1886.180 | 37.243 | 1923.4 |
| AC-SCG-8 | 1.164 | 1.032 | 2.207 | 2014.834 | 94.785 | 2109.3 |
| AC-SCG-9 | 0.675 | 0.628 | 1.687 | 1575.276 | 26.765 | 1602.0 |

V_p_ = Total Pore Volume, W_o_ = Volume of micropores, L_o_ = Average Micropore Size, S_mi_ = Microporous Surface, S_e_ = Non-microporous Surface

Table S5. Elemental composition (ultimate analysis) of the spent coffee grounds and activated carbon.

|  | **SCG** | **H-SCG** | **AC-SCG-8** |
| --- | --- | --- | --- |
| Ultimate analysis  C (wt% daf)  H (wt% daf)  N (wt% daf)  S (wt% daf)  O^a^ (wt% daf) | 51.2  6.4  2.6  0.1  39.8 | 51.6  6.8  2.1  0.1  39.5 | 83.9  1.1  0.5  0.1  15.2 |

daf: dry ash-free basis. ^a^ By subtraction
